# Frequency of Prescribing and Administering Premedication in Paediatric Surgical Patients at a Tertiary Care Hospital: An Observational Study

**DOI:** 10.7759/cureus.22347

**Published:** 2022-02-17

**Authors:** Ausaf A Khan, Durriya Raza, Muhammad Qamar-ul-Hoda

**Affiliations:** 1 Anaesthesiology, Aga Khan University Hospital, Karachi, PAK

**Keywords:** midazolam in paediactric surgery, preoperative anxiolysis, paediatric premedication, preoperative anxiety, premedication

## Abstract

Introduction: Preoperative period is a stressful event, especially for paediatric patients undergoing surgery. Stress may lead to the development of perioperative maladaptive behaviour, activation of stress responses, and susceptibility to postoperative infections. To alleviate preoperative stress, the use of a multimodal approach including preoperative pharmacological premedication in the ward is recommended. We conducted an observational study to determine the frequency of prescribing and administering premedication in paediatric surgical patients.

Methods: This three-month retrospective observational study was conducted in the main operating room of the Aga Khan University Hospital, Karachi, from October to December 2014. It included all paediatric patients (aged 1-16 years) coming for elective surgery. Patients’ preoperative forms and medical records were reviewed, and data recorded for written orders of premedication and the timing of administration of the premedication drug in the inpatient ward/surgical day care ward. A p-value <0.05 was considered statistically significant.

Results: This study included 125 paediatric patients. Premedication was not prescribed to 40% (50/125) patients. In these patients, drug and dose were properly mentioned in 98.7% (74/75) of cases while the route and time of administration were not mentioned in 26.6% and 12% prescription orders, respectively. The premedication drug was administered in 67 out of 75 patients (89.3%) by ward nurses as per prescription. The administration of premedication was documented in 95.5% patients, but the time was missing in 46.3% of cases.

Conclusion: A significant number of patients were not prescribed preoperative premedication by the anaesthetist. Moreover, the route and timing of administration of drug were not mentioned especially in cases when premedication was prescribed in the wards.

## Introduction

The preoperative period is a stressful event for most individuals undergoing surgery. This is especially true in paediatric patients and is related to a limited understanding of the nature of illness and the need for surgery in young children. Almost 50% of children show signs of significant preoperative fear and anxiety that can result in an uncooperative, upset child in the operating/anaesthetic room and can lead to the development of maladaptive behaviour such as emergence delirium, increased postoperative pain, night time crying, new-onset enuresis, separation anxiety, eating disturbances, apathy, withdrawal, and temper tantrums in the post-operative period [1-4]. Preoperative anxiety also activates stress responses, resulting in significantly increased levels of steroids and susceptibility to postoperative infections [3].

Separation from parents, especially for pre-school-age children, and induction of anaesthesia are considered the most stress-inducing perioperative phases. The literature suggests the use of multimodal approach to alleviate perioperative anxiety, as most effective, with either non-pharmacological or pharmacological methods, or both [5,6]. Oral midazolam (0.5-0.75 mg/kg, administered 30-60 min before the induction of anaesthesia), with its beneficial effects of anxiolysis, amnesia, and rapid onset of action, is the most commonly used benzodiazepine [2,5-7].

The first step in premedication is prescription and then its administration at an appropriate time. In our setup, the decision of whether a child requires a sedative premedication is usually made at the time of preoperative anaesthesia assessment either at the clinic or after admission to the hospital, by an anaesthesiologist. Our study aimed to find out the frequency of such prescription and its timely administration in the ward before surgery.

## Materials and methods

This retrospective observational study was conducted after obtaining an approval from the Aga Khan University Ethical Review Committee (approval number 3145-ANA-ERC-14). It was conducted in the main operating room (OR) of the Aga Khan University Hospital (AKUH), Karachi, from October 2014 to December 2014. All paediatric patients (aged 1-16 years) from the ward or surgical day care (SDC) unit coming for surgery to the main OR at AKUH from 8.00 am to 5.00 pm were included in the study. The preoperative assessment form and medical record of the study patients were reviewed, and the following information was recorded in the study proforma: (a) written orders for premedication with the drug, dose, timing, and route of administration and (b) the timing of administration of the premedication drug in the ward/SDC.

All data were entered, and statistical analysis was performed using IBM SPSS Statistics, version 19 (IBM Corp., Armonk, NY). Frequencies and percentages were computed for categorical outcomes and analysed by a chi-square test. Means and standard deviations were estimated by continuous data and analysed using Student’s t-test. A p-value <0.05 was considered statistically significant.

## Results

A total of 125 paediatric patients were included, with 68% from the SDC unit and 32% from the inpatient surgical ward, coming for elective surgery to the main OR. The average age and weight of the patients were 4.82 (SD 3.88) years and 19.06 (SD 13.38) kg, respectively; 81.6% were male and 18.4% were female.

Out of 125 patients, only 36% visited the preoperative anaesthesia assessment clinic while 60% were seen in the wards/SDC unit and 4% were assessed preoperatively at both places (Table 1). Premedication was prescribed to 60% of the patients. Quality of prescription was also evaluated, and it was observed that the drug and dose were properly written in 98.7% of cases while the route and time of administration were missing from 26.6% and 12% prescription orders, respectively.

**Table 1:** Prescription of premedication in paediatric surgical patients SDC, surgical day care

Premedication prescription	At the clinic, n (%)	In the ward/SDC, n (%)	At both places, n (%)
Patients undergoing preoperative anaesthesia assessment (N=125)	45 (36)	75 (60)	5 (4)
Premedication prescribed by the anaesthetist	31 (68.9)	41 (54.7)	3 (60)
Name of the drug mentioned in the prescription	31 (100)	41 (100)	2 (66.7)
Dose of drug mentioned in the prescription	31 (100)	41 (100)	2 (66.7)
Route of administration mentioned in the prescription	23 (74.2)	31 (41.3)	1 (13.3)
Timing of administration mentioned in the prescription	26 (83.9)	39 (52)	1 (13.3)

The premedication drug was administrated as per prescription in 89.3% of patients by ward nurses while in 10.7% of cases, it was not administered despite written orders. The administration of premedication was documented in patient’s medical record in 95.5% (64/67) cases, but the timing of administration was missing in 46.3% of cases (Table 2).

**Table 2:** Administration and documentation of premedication in paediatric surgical patients SDC, surgical day care

Premedication prescription	Prescribed at the clinic (n=31), n (%)	Prescribed in the ward/SDC (n=41), n (%)	Prescribed at both places (n=3), n (%)
Premedication drug administrated as per the prescription	28 (90.3)	36 (87.8)	3 (100)
Administration of premedication documented	27 (96.4)	34 (94.4)	3 (100)
Time of administration documented	17 (60.7)	16 (44.4)	3 (100)

In comparison, there were 31.1% (14/45) and 45.3% (34/75) patients who were not prescribed premedication by anaesthetists at the preoperative anaesthesia clinic and in the ward/SDC unit, respectively. Moreover, drug and dose were written as per standard, but the route and timing of administration were not mentioned as per standard especially in cases where premedication was prescribed in the wards/SDC unit.

## Discussion

In this study, the rate of prescription of premedication in paediatric patients was 60%. This was comparable to a study done in Sweden that reported the rate being 50%-60% [8]. The practice of prescribing anxiolytic premedication differs between institutions and is usually based upon the local patient population and departmental consensus [9]. The probable risk factors for perioperative anxiety and consequent morbidity are young age (between one and three years), anxious parents and previous bad or repeated anaesthetic encounters [3]. The trend of employing at least some form of premedication in anaesthesia practice has gradually increased over the years keeping in view the detrimental effects of preoperative anxiety in children and the improved behavioural outcomes associated with premedication [10]. The reported rate of prescription of sedative premedication is the highest (98.5%) among paediatric patients coming for congenital heart disease surgery [11].

On further sub-analysis of data, it was observed that premedication was prescribed more frequently to the patients who had their preoperative anaesthesia assessment done at the clinic than those who had it done in the ward or SDC unit. These findings of our survey are consistent with the previously reported literature that showed a similar rate of premedication prescription [11-14]. A possible reason for the higher prescription rate at the clinic can be the presence/direct supervision of the preoperative assessment and orders by the consultant anaesthesiologist at the clinic as opposed to the preoperative assessment done by the trainee in the ward as they might be unsure if premedication is indicated for that patient.

Another important finding of our study was that the drug and dose were properly documented on the prescription but the timing and route of administration of premedication were not documented in most of the premedication prescriptions. It is crucial to write the complete prescription whenever ordering any medication because incomplete prescriptions and dosing errors lead to the majority of prescribing errors [15].

Mentioning the time of administration of the drug is also essential since the timing of premedication is pivotal for it to effectively alleviate preoperative anxiety. Oral midazolam should be administered 30-40 minutes before moving the patient to the anaesthesia induction room/OR [3].

Our survey revealed that midazolam was the only drug used for premedication at our institute, despite other drugs for premedication being available as well. This was in accordance with the previous studies that showed midazolam as the most used drug to alleviate anxiety in children followed by fentanyl, dexmedetomidine and ketamine [2]. Studies have demonstrated that midazolam reduces preoperative anxiety, increases cooperation, and has minimal side effects, a short half-life, and good absorption after oral administration. The most common route of administration of premedication according to our study was oral. This was again comparable to the existing literature that depicts that most clinicians use the oral route (93%) [7].

Compliance for the administration of the drug as per the prescription was 89.3% in our patients. However, documentation was lacking in almost more than half of the cases. No previous literature was found to compare these study findings, but 100% compliance to the prescription should be the standard of care. The reasons for non-compliance were not sought out in our study and this aspect might be considered for exploration in future research on this subject. Another area worth investigating would be the short-term and long-term outcomes following non-prescription of premedication in this patient population. Based on our study, strict compliance to drug orders and proper documentation of administration is strongly recommended.

## Conclusions

Our study identified some inadequacies pertaining to the practice of premedication as well as documentation of drug administration. It was revealed that a significant number of patients were not prescribed preoperative anxiolytic premedication that might contribute to perioperative anxiety and adverse behavioural outcome. Some deficiencies in prescription writing were also highlighted, such as the route and time of administration were missing in most of the drug orders. The documentation of drug administration too was deficient. The findings of this study thus prompt measures to ensure strict compliance to drug prescription and documentation to improve patient outcome.
